# Beyond Tasks: Care Workers’ Perspectives on When Things Go Well in a Retirement Community Setting

**DOI:** 10.1093/geroni/igaf122.2685

**Published:** 2025-12-31

**Authors:** Tina Kilaberia, Daniel David

**Affiliations:** New York University, New York, New York, United States; New York University, New York, New York, United States

## Abstract

Care work in long-term care is demanding. This study asked 44 care workers about their perspectives of successful interprofessional practices to elicit an assets-based approach for the retooling and retention of workforce. Care workers delivered care on a continuum in independent living, assisted living, and a skilled nursing facility to approximately 700 residents daily. All were asked to reflect on and give examples with regard to the question: “when things work well, why do you think that happens?” The sample included professionals such as nurses, social workers, chaplains, rehabilitation specialists, dieticians, directors; and paraprofessionals such as certified nursing assistants, certified medication assistants, and environmental services workers. Two coders independently analyzed data with inductive coding first. Consensus was reached describing common themes on organizational, peer-to-peer, and resident levels. On the organizational level, communication and coordination were substantiated as positive aspects. On the collegial level, shared ethos (good heart, kindness, team spirit) was influential. On the resident level, person-centered focus stood out. Subsequently, reflecting this coding deductively through the Integrated Team Effectiveness Model (ITEM) showed greater influence of the process elements of communication and coordination, and secondary influence of the related external context (regulatory framework, accountability to families). Task-related factors were not identified as influential to working well. This suggests that in residential settings, creating effective care environments may depend more on linking organizational processes, human factors, and a resident-focused culture as components of teamwork. This has implications for the approach to workforce development and retention in long-term care.

